# Development of the Health Atlas of Jalisco: A New Web-Based Service for the Ministry of Health and the Community in Mexico

**DOI:** 10.2196/publichealth.5255

**Published:** 2016-03-16

**Authors:** Igor Martin Ramos Herrera, Miguel Gonzalez Castañeda, Juan Robles, Joel Fonseca León

**Affiliations:** ^1^ Center of Research on Geographic Information Systems and Management in Health Department of Public Health University of Guadalajara Guadalajara Mexico; ^2^ Center of Social Sciences and Humanities Department of Geography and Territorial Ordering University of Guadalajara Guadalajara Mexico; ^3^ Statistics analysis area Statistics Department Mexican Institute of Social Security Guadalajara Mexico

**Keywords:** Public health, health atlas, geographic information systems, geographic mapping, online systems, health information systems

## Abstract

**Background:**

Maps have been widely used to provide a visual representation of information of a geographic area. Health atlases are collections of maps related to conditions, infrastructure or services provided. Various countries have put resources towards producing health atlases that support health decision makers to enhance their services to the communities. Latin America, as well as Spain, have produced several atlases of importance such as the interactive mortality atlas of Andalucía, which is very similar to the one that is presented in this paper. In Mexico, the National Institute of Public Health produced the only health atlas found that is of relevance. It was published online in 2003 and is currently still active.

**Objective:**

The objective of this work is to describe the methods used to develop the Health Atlas of Jalisco (HAJ), and show its characteristics and how it interactively works with the user as a Web-based service.

**Methods:**

This work has an ecological design in which the analysis units are the 125 municipalities (counties) of the state of Jalisco, Mexico. We created and published online a geographic health atlas displaying a system based on input from official health database of the Health Ministry of Jalisco (HMJ), and some databases from the National Institute of Statistics and Geography (NISGI). The atlas displays 256 different variables as health-direct or health-related indicators. Instant Atlas software was used to generate the online application. The atlas was developed using these procedures: (1) datasheet processing and base maps generation, (2) software arrangements, and (3) website creation.

**Results:**

The HAJ is a Web-based service that allows users to interact with health and general data, regions, and categories according to their information needs and generates thematic maps (eg, the total population of the state or of a single municipality grouped by age or sex). The atlas is capable of displaying more than 32,000 different maps by combining categories, indicators, municipalities, and regions. Users can select the entire province, one or several municipalities, and the indicator they require. The atlas then generates and displays the requested map.

**Conclusions:**

This atlas is a Web-based service that interactively allows users to review health indicators such as structure, supplies, processes, and the impact on public health and related sectors in Jalisco, Mexico. One of the main interests is to reduce the number of information requests that the Ministry of Health receives every week from the general public, media reporters, and other government sectors. The atlas will support transparency, information diffusion, health decision-making, and the formulation of new public policies. Furthermore, the research team intends to promote research and education in public health.

## Introduction

Information in health has become an asset for governments and institutions in many countries. Registers of health and disease events, inventories of health resources and infrastructure, medical records, and registers of preventive actions are only a few of the many sources of health data [1,2]. In the last twenty years, a lot of work has focused on the use of Geographic Information Systems (GIS) around the world [3]. These systems are defined as computer systems capable of assembling, storing, manipulating, and displaying geographically referenced information (ie, data identified according to their locations) [4]. However, their use in health information improved when they were used to support decisions on service implementation, epidemic monitoring, and public health interventions [5]. One of the applications of these systems is the generation of atlases, which are sophisticated tools capable of managing, storing, and showing spatial relationships. Atlases can be printed on paper, but are also available in electronic or Web-based, interactive or multimedia formats. In health, research has described the distribution of health actions and disease cases from a geographic standpoint, using aggregated information at country, state or county level, whether raw numbers or standardized rates [6]. Health atlases are a collection of maps related to health conditions which provide a unique method to analyze data and describe the magnitude of health problems, identify their relationships with social situations, determine conditioning factors, and support decision making from health authorities, government, non-government organizations, and the community.

The use of maps to show health information provides a visual representation of the distribution of a health situation or condition and its relation to many other environmental, dietary, and physical infrastructures, human resources, among many others [7]. For example, a geographic atlas was used in Spain to show mortality information that guided health authorities to focus their services where they were needed the most [8]. Another atlas was used in Taiwan to report mortality rates on oral cancer related to environmental risks [9], where they found a spatial correlation between the presence of some metals in the environment and the incidence of oral cancer. Another example is the Atlas of Reproductive Health that the Center for Disease Control (CDC) of Atlanta released in 2004 to show demographic and risk factors related to this topic [10]. More recently, a Web-based system was developed in Saudi Arabia to manage registry data for patients with diabetes [11].

In Latin America, there have been several efforts to develop health atlases from different perspectives and areas, such as the atlas of cancer mortality in Colombia, which shows the distribution of cancer mortality in a series of maps according to gender and state [12]. The Health Atlas of Mexico that was made in 2003, displays health and disease distributions according to state and counties, and was published in compact disc (CD) and in a Web-based platform [13]. The Web-based atlas developed at the Pan-American Health Organization in 2003 [14], and some other atlases devoted entirely to show the health conditions of specific states in one country, such as the one we report here, have been developed [15,16]. All of them served their respective ministries to make health-related decisions and take the corresponding actions with different results.

Since 2013, the government of Jalisco established a new law called *Law of Transparency and Public Information Access*, which compels all public offices to publish their operations and economic and management information related to the use of public resources [17]. This law also establishes that any citizen can request information from any state office and the officials must answer promptly or be sanctioned for the delay. For this reason, in 2014, the Health Ministry of Jalisco (HMJ) signed an agreement with the research team from the University of Guadalajara responsible for the project reported in this paper to provide information, and use this atlas as one of its main sources of information for purposes of the Transparency Law.

The interactive Health Atlas of Jalisco (HAJ) is a service created by the University of Guadalajara, Mexico, through a grant by the Council of Science and Technology of the Province of Jalisco. The HMJ provides approved information to feed the system and publishes it as a Web-based service. The aim of the service is to support health decision makers and inform media reporters and the general public with a collection of thematic maps that represent the main health indicators of the Province of Jalisco, based on data from 2010 to 2013. In addition, it serves to indicate the status of the health-related Millennium Development Goals established by the United Nations (UN) in the state [18]. Thus, the objective of this work is to describe the methods used to develop the HAJ, as well as show its characteristics and how it interactively works with the user as a Web-based service.

## Methods

A multidisciplinary research team comprised of a physician, geographer, mathematician, and statistics specialist all working at the University of Guadalajara developed the HAJ. In total, the design and development of the HAJ took about two and a half years, which includes the time spent in making arrangements with the HMJ and the National Institute of Statistics and Geography (NISGI, INEGI in Spanish), the organizations that provided the data used as input to the system. The digital maps are generated using the Instant Atlas software [19], which creates an interactive, online tool for the end user to manipulate and display the atlas data as digital maps. To achieve this, the team generated the website, manipulated the input data, prepared the base maps, and gave the instruction set so the software could process the data and generate the mapping tool.

This work is based on an ecologic design in which the analysis unit was the municipality (county) of the state of Jalisco in Mexico. We created and published online a Geographic Health Displaying System in which we used HMJ´s official health database and databases from the NISGI as input to complete the project. For this purpose, we acquired a license for the Instant Atlas software as our base program to generate the interactive online application [19].

### Scope

Jalisco is one of the 32 states (departments) of Mexico. It is located in the Central Western area of the country and it is the 7th biggest state in Mexico with an area of 78,588 km^2^. Jalisco has 125 municipalities (counties), a population of more than 7.3 million people distributed in urban (87%) and rural (13%) areas, and ranks as the 4th most populated state in the country (6.5%). There are 3,750,041 women (51%) and 3,600,641 men (49%) in Jalisco, with a mean age of 25 years. In 2013, the gross domestic product of the state represented 6.4% of the country’s total.

### Analysis Units

While the main scope of the atlas is the state of Jalisco, its analysis units are the municipalities. There are 125 municipalities in the state with a mean distribution of 93.5 inhabitants per km^2^. The municipality with the largest population is Guadalajara with 1,495,189 inhabitants on a surface of 187.91 km^2^, only 0.23% of the state’s surface. The least populated municipality is Ejutla with only 2082 inhabitants on a territory of 472.21 km^2^, representing 0.60% of the state’s surface. Ejutla has three times more surface than Guadalajara and a population density of 4.42 inhabitants per km^2^, while Guadalajara´s density is of 7957 inhabitants per km^2^. Thus, a great disparity in population distribution exists in Jalisco [20]. More than 50% of the population lives in only five municipalities: Guadalajara, Zapopan, Tlaquepaque, Tonalá (central region), and Puerto Vallarta (north coast region), with 1.49 million inhabitants in Tonalá and only 250,000 in Puerto Vallarta.

### Variables

The atlas is divided into seven broad, health-related themes, where three of them (mortality, morbidity, and health infrastructure) are direct health indicators and four of them (demography, socio-economy, education, and living condition) are general health-related indicators. Each of the seven themes are composed of different health indicators (referred to as variables in the atlas) and broken down as follows: (1) demography (36 variables), (2) socio-economy (18 variables), (3) mortality (43 variables), (4) morbidity (59 variables), (5) health infrastructure (3 variables), (6) education (18 variables), and (7) living conditions (79 variables). Each variable generates a thematic map resulting in seven thematic maps and a total of 256 variables.

Most of the variables are represented in absolute numbers while some are shown as percentages, and a few as rates. The variables are not all represented in the same format because they were received them from the HMJ and the NISGI, and because we did not want to change the data so it reflected the real situation of the state according to those institutions.

There are two major regional divisions in the HAJ: the 12 sanitary regions made by the HMJ, and the 13 planning regions made by the Jalisco´s State Planning Office (JSPO, COPLADE in Spanish). The difference is because the HMJ divided the state more than twenty years ago for health service delivery purposes, whereas the Government of Jalisco, through the JSPO, divided the state about fifteen years ago for planning purposes. The regions of both divisions have different municipalities; therefore the results given by the atlas are different too. Given that, the user needs to verify which major division to select in order to receive the adequate information.

### Information Sources

The information regarding health indicators was provided by the HMJ, who provided databases in datasheet format corresponding to mortality, morbidity, and health infrastructure of the state, divided by municipalities for the year 2013. In addition, information was extracted by mining the NISGI website [20], and demographic, socio-economic, education, and housing information corresponding to the 125 municipalities for the year 2013 was obtained. The information from both sources was retrieved in datasheet format and then manipulated to feed the GIS used in this project.

### Procedures

#### Datasheet Processing and Base Maps Generation

**Figure 1:**
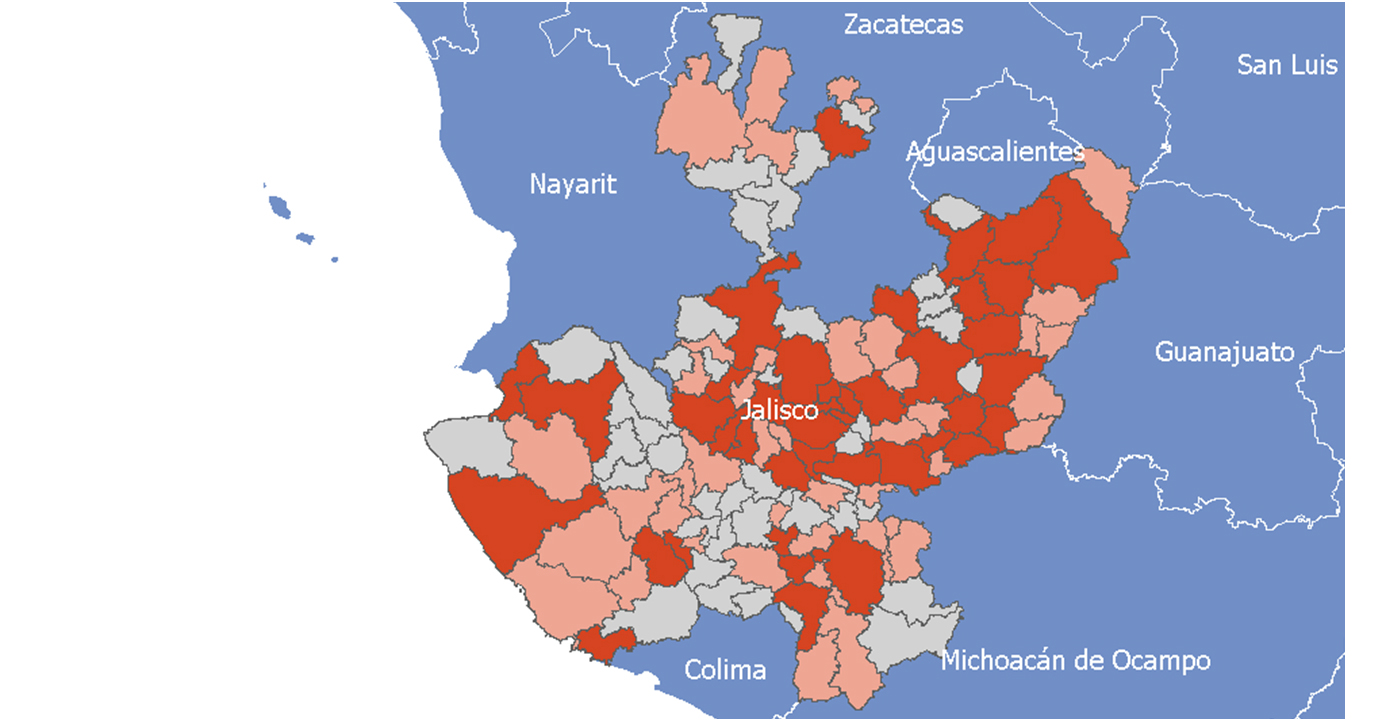
Example of a data shape of the state of Jalisco, Mexico.

Using the coding system provided by the NISGI in which each of the 125 municipalities has a unique code or number, we processed the datasheets received from the HMJ (118 health indicators) and collected from the NISGI website (138 general indicators). The 256 indicators were then processed as variables in datasheet format according to software specifications in order to generate the same number of tables that served as input. Each municipality was also assigned two region numbers according to the twelve sanitary regions and the thirteen JSPO regions that appeared as new variables on each table created (Multimedia Appendix 1). Three different base maps (data shapes) were then generated to be used in all the analytical categories, so we could combine them with the HMJ and NISGI’s datasheets (Figure 1). The shapes were the maps of the territory analyzed according to its real geographical coordinates defined by the NISGI and also obtained through its website. The shapes were processed according to the project’s specific needs and then prepared for the requirements of the software. The shapes are polygonal databases that save the data of the entire studied territory, in this case the state of Jalisco, and of all the municipalities within one registry (row) for each municipality in the "municipality code" field. Thus, each municipality was assigned to a row on the shape database and then the corresponding information of each one was captured on the same row, which would later be processed inside the software to generate the thematic maps.

#### Software Arrangements

The data files were processed offline using Instant Atlas software [19] to combine data shapes and datasheets and generate the online files for the users. The software is capable of generating every necessary file to publish the map on the Web. For example, in the mortality category, the software generated more than 50 files that included the datasheet file (csv and xslt), the shape files (js), the image files (PNG, GIF, and JPEG), and the html files (html and css). The generated files were ready to upload into the Web server with no further manipulation,

#### Making Up the Website

The goal of the HAJ is to provide an open access, free service for the HMJ, health related decision makers, non-government organizations, and the community. As such, we generated a free, searchable website accessible through the Internet. The grant we received from the Council of Science and Technology of Jalisco allowed us to pay for the hosting and the URL name for two years.

Two hosting providers were contracted. The first was Wix that was used to prepare the overall interface of the project with general information about the research project, the research team, tutorials, and credits of the university and the collaborating and granting institutions. On this site, one section to publish the atlas was prepared but no files of the atlas were saved on this server. Instead, we made a link where the files were stored. The second is where we hosted and organized the files generated by the GIS software on the other contracted server where the link on the first server pointed. A local provider manages this server for security (check that all the files are secured and protected) and accessibility (managing the website) purposes. The contracts of both hosting services included the payment of the URL names for the site, so we did not have to pay for them separately. In 2014, we uploaded the generated files into the server and set the online version of the HAJ as well as the URL given, so it could be available for user review.

## Results

### General Characteristics and Technical Specifications of the HAJ

The HAJ is a Web-based service that provides a friendly environment for users to interact with health and general data, regions, and categories according to their information needs; the atlas generates thematic maps about the situation of the state for each health indicator and category requested (ie, the total population of the state or of a single municipality, grouped by age or sex). It is capable of generating and displaying more than 32,000 different maps by combining categories, indicators, municipalities, and regions, so users can select the entire province, one or several municipalities, and the indicator that they require. Then, the atlas generates and displays the requested map. Users can then print it or save it in PDF, spreadsheet, PNG or html formats. This represents a very large amount of information for the health ministry and the community of the state. Access to the atlas can be found on its website [21].

### Selection of a Thematic Map

**Figure 2:**
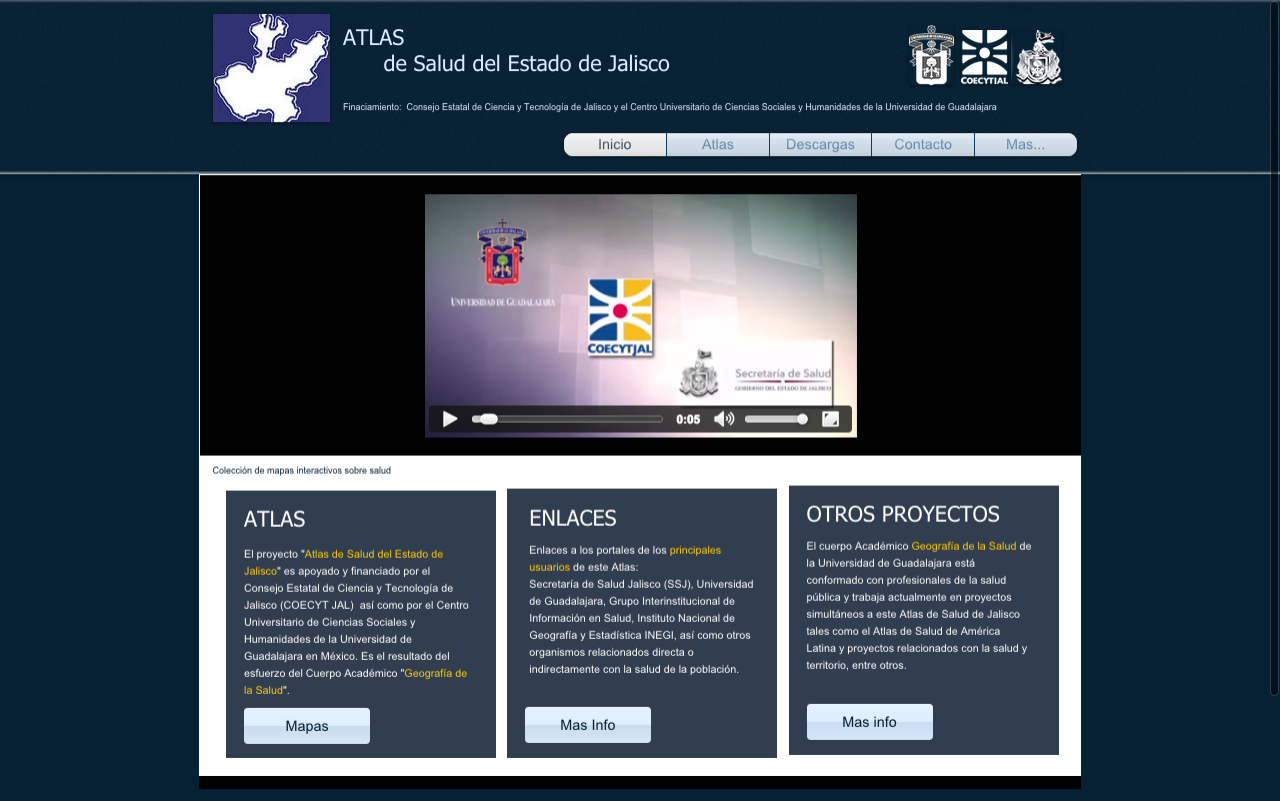
Homepage of the Health Atlas of Jalisco showing the main menu and the introductory video.

The main page of the HAJ (Figure 2) shows an introductory video that explains why the atlas was made and what it can be used for. The video plays automatically when the webpage is opened, but it can be paused or stopped at any time, shown in full screen or in the default small-frame setting. The logos of the institutions involved in the project and the grant credits are shown at the top of the page. The main menu is displayed under the logos. The five main menu options are Homepage (*Inicio*), Theme Selection (*Atlas*), Downloads (*Descargas*), Contact (*Contacto*), and More Info (*Más*). Each link takes the user to a different area.

To see the thematic maps users must get to the mapping tool. This can be done by following two paths. The first is selecting the Atlas option on the main menu that opens the Theme Selection page, which shows the previously mentioned categories and a general explanation of each. If a category is selected, the mapping tool on that category is displayed. The second is selecting a category from the submenu in the Atlas option on the main menu, which links directly to the mapping tool to choose data from the category selected.

### The Mapping Tool

The site includes a tutorial on how to use the mapping tool; users must select the "More Info" option of the main menu and select the "Extras" option from the submenu. There, the "Video Tutorial" link opens an 8-minute video clip that explains how to use the mapping tool.

### Interactive Characteristics

**Figure 3:**
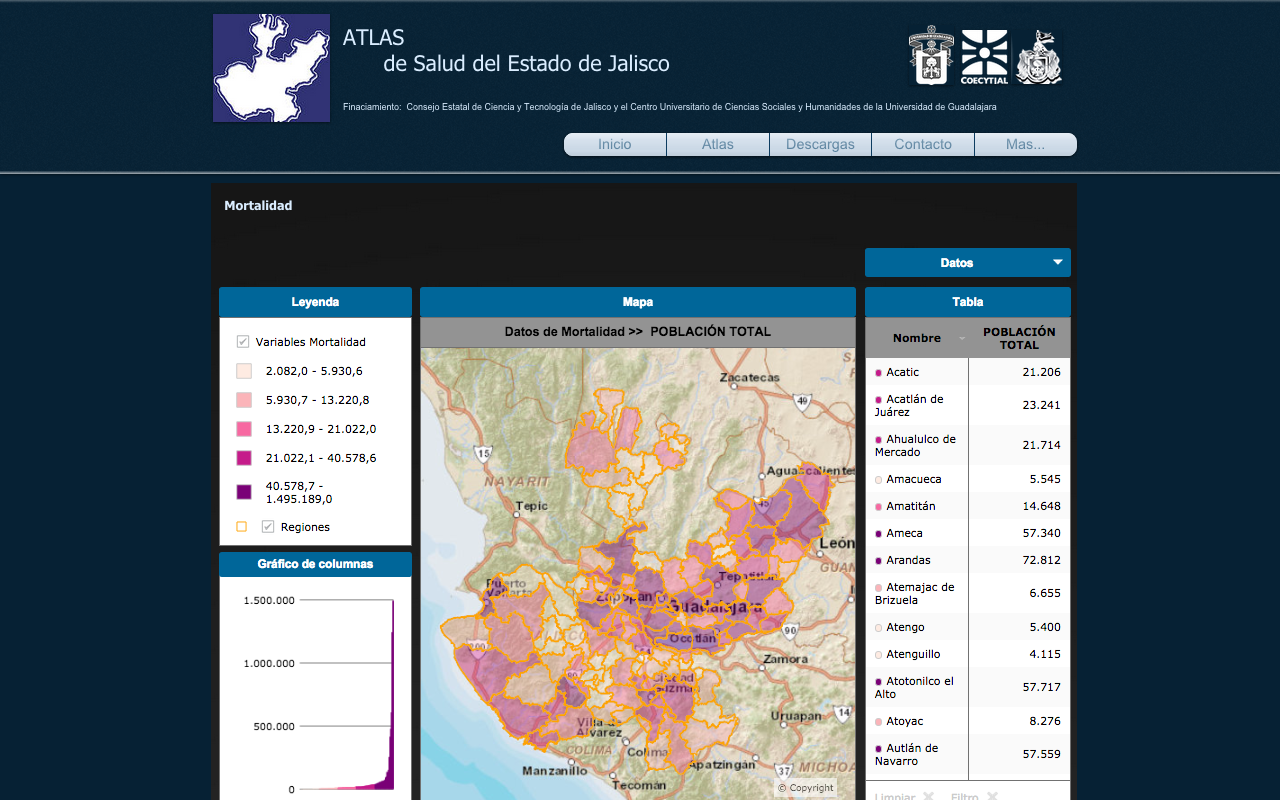
The mapping tool of the Health Atlas of Jalisco.

Whether the user selects the first or second path, the site directs him/her to the mapping tool. The mapping tool with the *Mortalidad* (mortality) category in the upper left corner of the screen is shown in Figure 3. There are four frames in the center of the screen. The mapping tool will display information on any element within the frame simply by moving the mouse over that element. Clicking on any element, however, produces a change in the map in the middle, as explained in Textbox 1.

**Description of the frames in the mapping tool.**

Frame and description

1. The *Leyenda* (Legend) frame activates or deactivates the four layers displayed on the map (variables, regions, municipality limits, and the world street map) just by clicking their name on the Legend frame.
2. The *Gráfico de Columnas* (Bar Graph) frame shows a bar graph according to the variable selected, if any bar is selected it activates the corresponding region or municipality on the map.
3. The *Mapa* (Map) frame in the middle displays the map of Jalisco according to the variable and layer selected, and shows the quantification of the municipalities in color data groups. If the mouse is placed over a municipality, it shows its name and value, but if it is clicked, the map highlights its geographical limit here as well as in the other frames. In addition, the frame has a small box on the top left corner that allows the user to zoom in and out of the state, reset it to its original size or clean any selected filter.
4. The *Tabla* (Table) frame shows the names and values of all the municipalities of the state in two columns. If the mouse is placed on any of them, the atlas will display it on the map frame; but if it is clicked, the frame will highlight it and the map will zoom in on that municipality to take a closer look at it and the surrounding areas. In addition, the frame allows the user to scroll up and down to reach all the 125 municipalities of the state.
5. The last element of the mapping tool is the *Datos* (Data) button. When clicked, this button displays a new box that allows the user to select the type of region, sanitary or planning that will be displayed, and the category’s variable data that will be displayed on the other four frames.

#### Data Retrieval per Health or Planning Regions

The aim of the mapping tool is to help users find information about a particular indicator of the health situation in the state of Jalisco. Thus, the first step is to select a category or theme from the Atlas menu (eg, mortality). Whenever a category is selected, the data set changes on the mapping tool, but this is not visible until the user clicks on the Data button where the data changes according to the category selected. The second step is to select a type of region by clicking on the Data button, and selecting *Regiones COPLADE* or *Jurisdicciones Sanitarias*; this action changes the elements on the Legend frame. The third step is to select a variable from the data box. In the case of the mortality category, it appears as *Datos de mortalidad* (mortality data), which displays a list of 43 variables that the user can select (eg, traffic accidents or diabetes). If a variable is clicked, the contents of all the other frames change to display data on that specific variable. Finally, the user can move over the map, legend, and graph bar frames to select the information required as needed.

#### Map Download and Visits

The mapping tool is not yet able to generate an output by itself, but the research team is currently working to provide a list of maps in PDF format of almost all the 256 indicators currently available on the website [22]. This map list will be available in the *Descargas* (Downloads) section of the main menu. Today, if a user wants a selected map that is not available in that section, he/she has to generate it with the mapping tool and then take a screenshot to obtain an image of it.

To date, the HAJ has received more than 1400 visits, most of them from HMJ health authorities that are testing the atlas function. In addition, there is a small visit counter on the main page just for information purposes; however, the hosting service provides detailed information of the visitors and their characteristics that will allow the team to identify them and the information they check in order to improve the service with time.

The research team is now engaged in analyzing the statistics reported by the HMJ and generating the maps of two separate years, 2000 and 2013. This will serve to compare and show any changes in the UN's millennium goals. We will also check how every municipality and health jurisdiction is doing. This aspect of the project should be complete by December 2015.

## Discussion

### Principal Findings

Here, we described how the HAJ was made and how it functions. The HAJ is a Web-based service that interactively allows users to review health indicators such as structure, supplies, processes and the impact on public health and related sectors, in order to request and identify opportunity areas and good practices in health services in Jalisco, Mexico. For example, users may use it to analyze the distribution of vaccines in the state and then make projections by age groups or life dimensions about the needs of the population for general care [23].

The atlas is intended to help the HMJ in (1) reducing the volume of information requests from the health sector of Jalisco; (2) maintaining available data from multiple sources with a different grouping or presentation; (3) presenting in map format a considerable volume of data for users with different interests; (4) unifying health data focalization criteria; (5) facilitating graphic and cartographic data representation; (6) integrating the new technologies for request consultation; and (7) supporting the consultation of data for health decision making.

One of the main interests of the HMJ is to reduce the number of information requests that the Ministry of Health receives every week from the general public, media reporters, and other government sectors. These requests are made through the transparency channels and the ministry is forced by law to answer fast and correctly. The atlas will support transparency, information diffusion, health decision-making, and the formulation of new public policies. Furthermore, the research team intends to promote research and education in public health.

Showing health information in map format helps us to analyze the spatial relations between health and geographic situations (eg, mountains, rivers, and water bodies) that can affect the area accessibility. This action cannot be easily done just by looking at a graphic chart or a table. These topographic elements can sometimes represent important barriers to the resources sent to an area and can represent a delay of hours or even days to reach some communities for the health personnel because they have to travel in small planes or helicopters, by walking or riding a horse.

In addition, displaying the health situation in a map can help the authorities to make decisions on a sanitary region or municipality that are more or less affected by a disease. This can be done, for example, by putting over a transparent layer with financial information or human resources that are closer to the areas in trouble. Finally, spatial-temporal analyses can show how conditions can change over time to find tendencies or future perspectives.

### Limitations

Although we tried to gather the main health indicators, it is very difficult to have all of them because of confidentiality issues. The HMJ is very careful about releasing health data, in order to protect its users and personnel from information misuse or abuse. We were very clear on this matter and we only published a data collection from official sources and after receiving the authorization from the Ministry of Health.

Moreover, the service is not yet finished because users cannot generate an output map in PDF or JPEG format directly from the mapping tool. Instead, we created the Download section to make some maps available, but they may not comply with user needs. We will continue to work on these issues.

Another limitation is the level of aggregation. Here, we worked at the municipality level but the basic geographical-statistical areas (AGEB) level or blocks would be better. However, we are not sure if the data records at the HMJ are complete at those levels. In a second version of the atlas we are planning to work at the AGEB level, but then we will work with NISGI´s records. They make a population census every five years that gathers information at the house level, and we will try to reach that level.

### Comparison with Prior Work

Different researchers have studied the use of GIS and/or Web-based maps and its usefulness has been proved as an accepted method to provide information to users and to support health decision makers in providing a better service [24]. As Wong and colleagues [25] have demonstrated, GIS have important implications for improving public health; they developed a system that keeps track of public water sources to make decisions on managing water systems. Though their system was not specifically an atlas, it provides geographic information through maps that show water system boundaries and support authorities in decision-making.

In Latin America and Spain, most of the atlases have been developed to analyze the mortality situation of the respective countries. Ocaña and colleagues [8], for example, developed the interactive Atlas of the Andalucía region in Spain. This atlas is very similar to the one we created in that it displays a series of maps focused on mortality rates from that region, and compares them to the rates of the entire country. It is available on the Web and intends to help evaluate interventions and identify territorial inequities in resource distribution. It was reported as the first interactive mortality atlas in Spain.

However, Colombia [12] and Bolivia [15] each developed health atlases that show not only mortality information, but also health indicators in general. They show health, social, and economic information in order to provide an integral perspective of the health situation in those countries as well as indicate the problems shared by several regions or municipalities. Brazil also published its atlas of human development; they published it online, and it shows health information on a municipal scale [26]. This atlas is based on the human development index of the UN and it is intended to evaluate the effect of public policies on the population due to the country’s international agreements and future perspectives. Of note, other atlases available on the Internet are also based in Instant Atlas software [19].

### Conclusions

The HAJ was created to quickly review the profiles and tendencies in which health conditions, disease, and death indicators are distributed in the state as well as the services provided in different socio-economic contexts, geographical borders, and political and administrative limits.

These methods have been used recently to support health decision makers and inform the general public. Now, more institutions will be using maps to represent health situations because of its proximity to the analyzed areas [27], especially if researchers plan to use spatio-temporal, and cluster analyses [28].

As Iñiguez and Barcellos say [29], it is time for health geographers to start analyzing the health situation of population groups in a state or a region, which will help authorities to identify the distribution of problems according to environmental and life condition inequities [30]. Indeed, the next step of the research team is to address this.

## Appendix

Multimedia Appendix 1 Variable (indicator) coding system according to regions and categories.

## Abbreviations

AGEB: Basic Geographic-Statistical Areas
GIS: Geographic Information Systems
HAJ: Health Atlas of Jalisco
HMJ: Health Ministry of Jalisco
JSPO: Jalisco State’s Planning Office
NISGI: National Institute of Statistics and Geography
UN: United Nations
